# Supplementary figures and images for: HMGB1 amplifies ILC2-induced type-2 inflammation and airway smooth muscle remodelling
Source: PLoS Pathog. 2020 Jul 13;16(7):e1008651. doi: 10.1371/journal.ppat.1008651 (PMC7377495; doi:10.1371/journal.ppat.1008651)

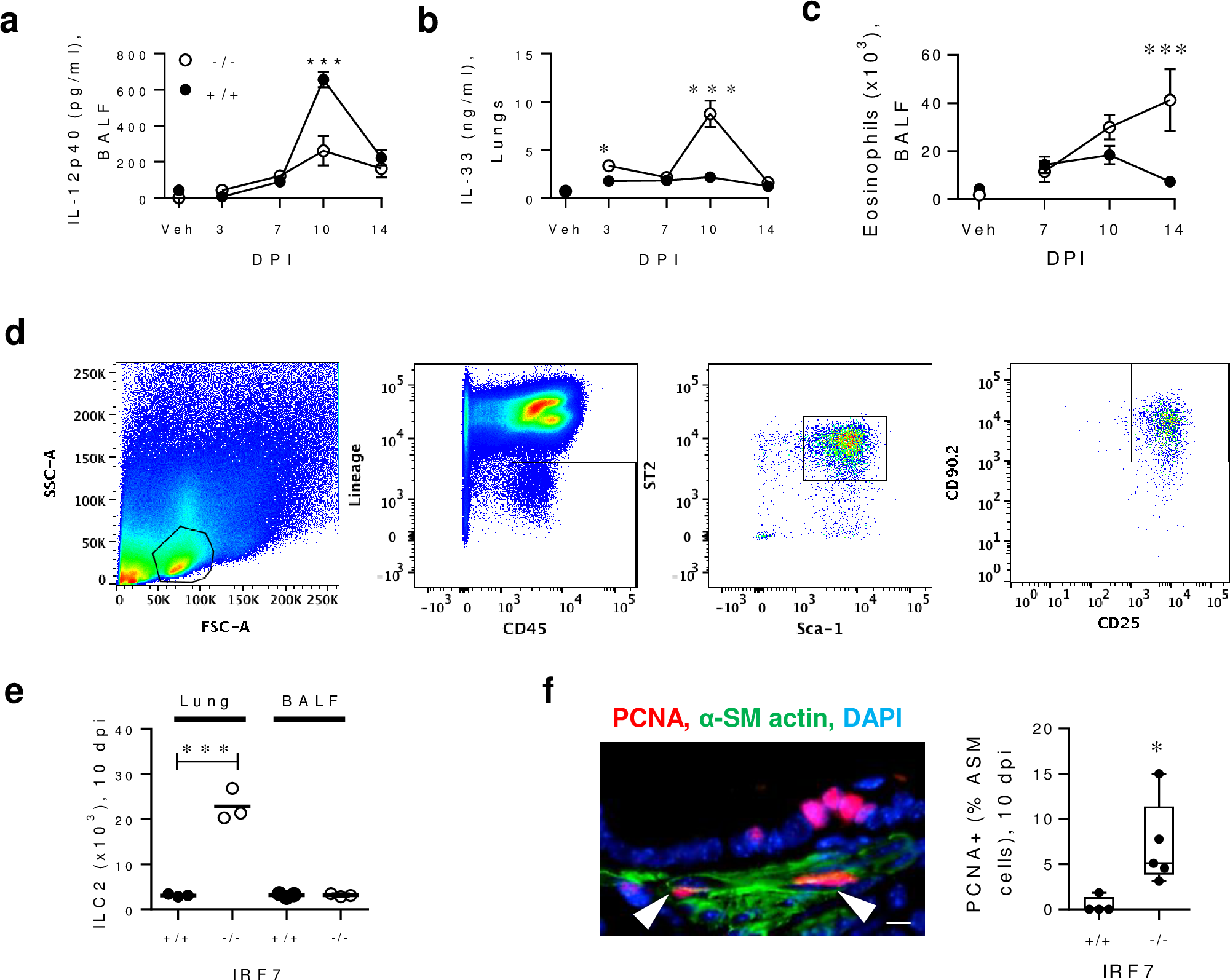

Supplement: S1 Fig — WT (IRF7+/+, closed circle) and IRF7-/- mice (open circle) were inoculated with PVM or vehicle at postnatal day 7 and samples collected at 3, 7, 10 and 14 days post infection (dpi). (a) IL-12p40 protein expression in BALF. (b) IL-33 protein expression in lungs. (c) Eosinophils in BALF. (d) Gating strategy for ILC2s: Lineage- (CD45R, CD3, CD4, CD11c, CD19, Gr-1, CD11b, CD2, NK1.1, CD49b), CD90.2+, CD45+, CD25+, ST2+, Sca-1+ ILC2 cells. (e) Numbers of ILC2 cells in left lung lobe and BALF in WT and IRF7-/- mice at 10 dpi. (f) Representative micrograph (x1000 magnification) of PCNA immunoreactivity (red) and smooth muscle actin immunoreactivity (green) in the lung at 10 dpi (left), quantified as % of ASM cells (right). Scale bar = 5 μm. Data are representative of n = 2 experiments with 3 to 8 mice in each group and are presented as mean ± SEM (a-c) or scatter plot (e) or as box-and-whisker plots showing quartiles (boxes) and range (whiskers; f). Data were analysed by Two-way ANOVA with Tukey post hoc test (a-d) or one-way ANOVA with Dunnett post hoc test (f) or T-test (g); *, P < 0.05; **, P < 0.01; ***, P < 0.001 compared with the WT control group. (TIF) [file ppat.1008651.s001.tif]

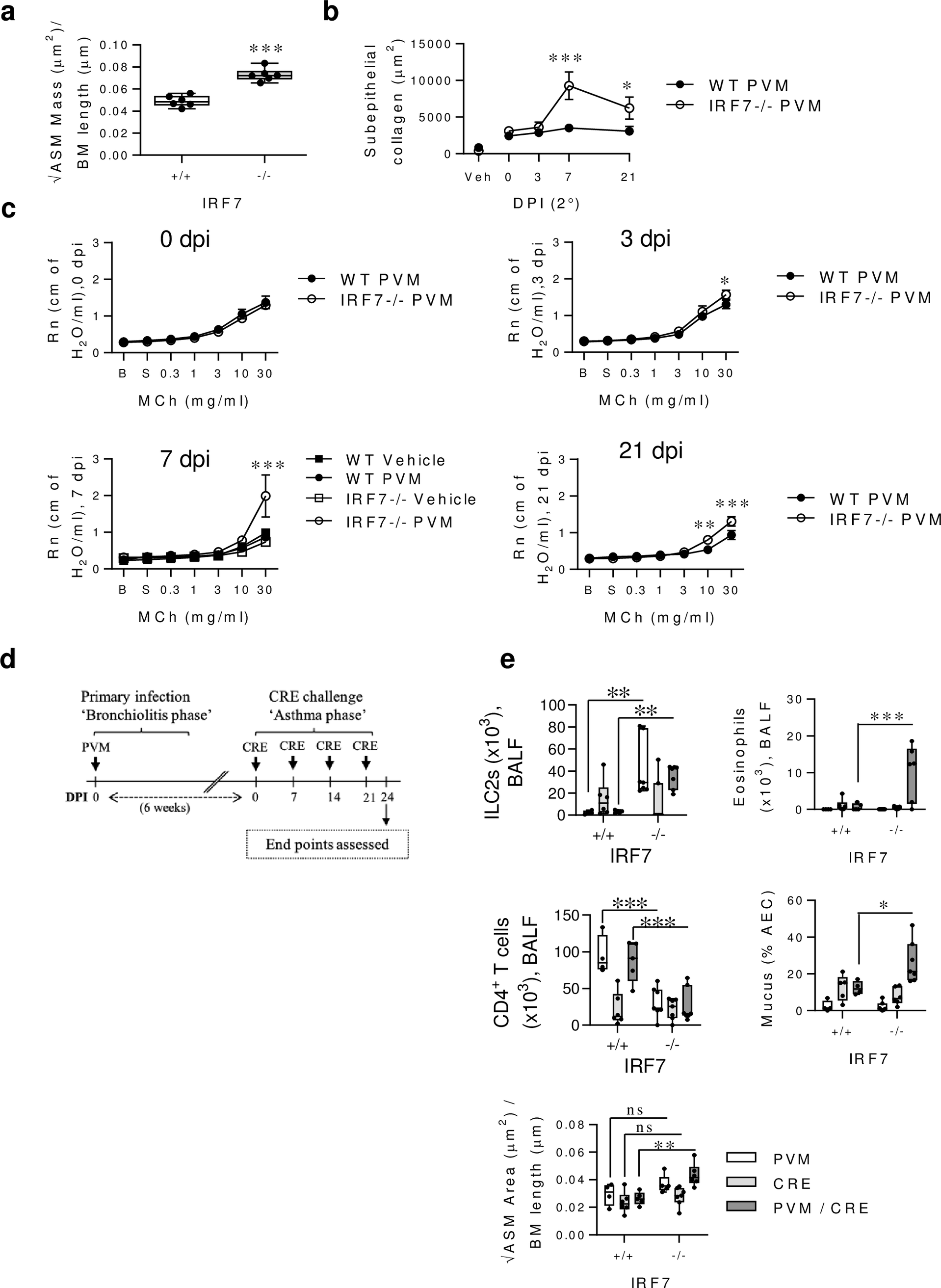

Supplement: S2 Fig — (a) Six weeks after the primary infection, WT (IRF7+/+) and IRF7-/- mice were re-infected with PVM and ASM area assessed 8 weeks later. (b) Subepithelial collagen deposition and (c) AHR at 0, 3, 7 and 21 days post secondary infection. (d) Study design. WT (IRF7+/+) and IRF7-/- mice were either inoculated with PVM at postnatal day 7 or exposed to cockroach allergen (CRE) 6 weeks later or both. (e) Total ILC2 (Lineage- (CD45R, CD3, CD4, CD11c, CD19, Gr-1, CD11b, CD2, NK1.1, CD49b), CD90.2+CD45+CD25+ST2+Sca-1+), eosinophil (Siglec F+CD11b+Ly6GintMHCII-CD3-B220-) and CD4+ T cells (CD3+ CD4+ CD8-) numbers in BAL were identified by flow cytometry, followed by mucus-producing airway epithelial cells (AECs) and ASM area quantification. Data are representative of n = 2 experiments with 4 to 8 mice in each group and are presented as box-and-whisker plots showing quartiles (boxes) and range (whiskers; a, e) or as mean ± SEM (b-c). Data were analysed by T-test (a) or Two-way ANOVA with Tukey post hoc test (b-c) or one-way ANOVA with Dunnett post hoc test (e). *, P < 0.05; **, P < 0.01; ***, P < 0.001 compared with the WT control group. (TIF) [file ppat.1008651.s002.tif]

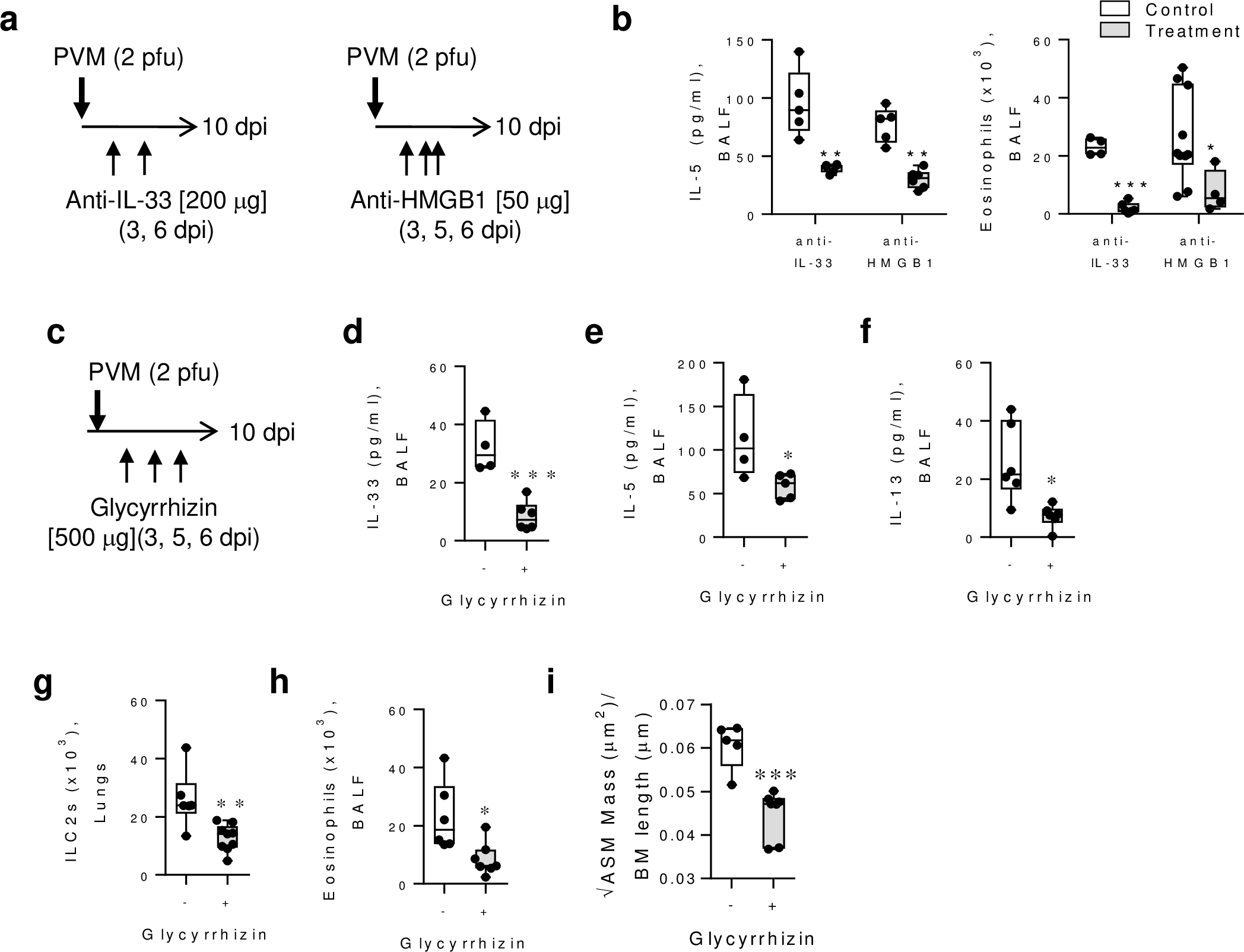

Supplement: S3 Fig — (a) Study design. (b) IL-5 expression in IRF7-/- mice in BALF (left) and eosinophils in BALF (right). (c-i) Effect of glycyrrhizin on type-2 inflammation in PVM-infected IRF7-/- mice. (c) Study design. (d) IL-33, (e) IL-5 and (f) IL-13 protein expression in IRF7-/- mice in BALF at 10 dpi. (g) ILC2s in lung. (h) Eosinophils in BALF. (i) ASM area. Data are representative of n = 2 experiments with four to eight neonates in each group and are presented as box-and-whisker plots showing quartiles (boxes) and range (whiskers; b, d-i). Data were analysed by T-test; *, P < 0.05; **, P < 0.01; ***, P < 0.001 compared with the IRF7-/- control group. (TIF) [file ppat.1008651.s003.tif]

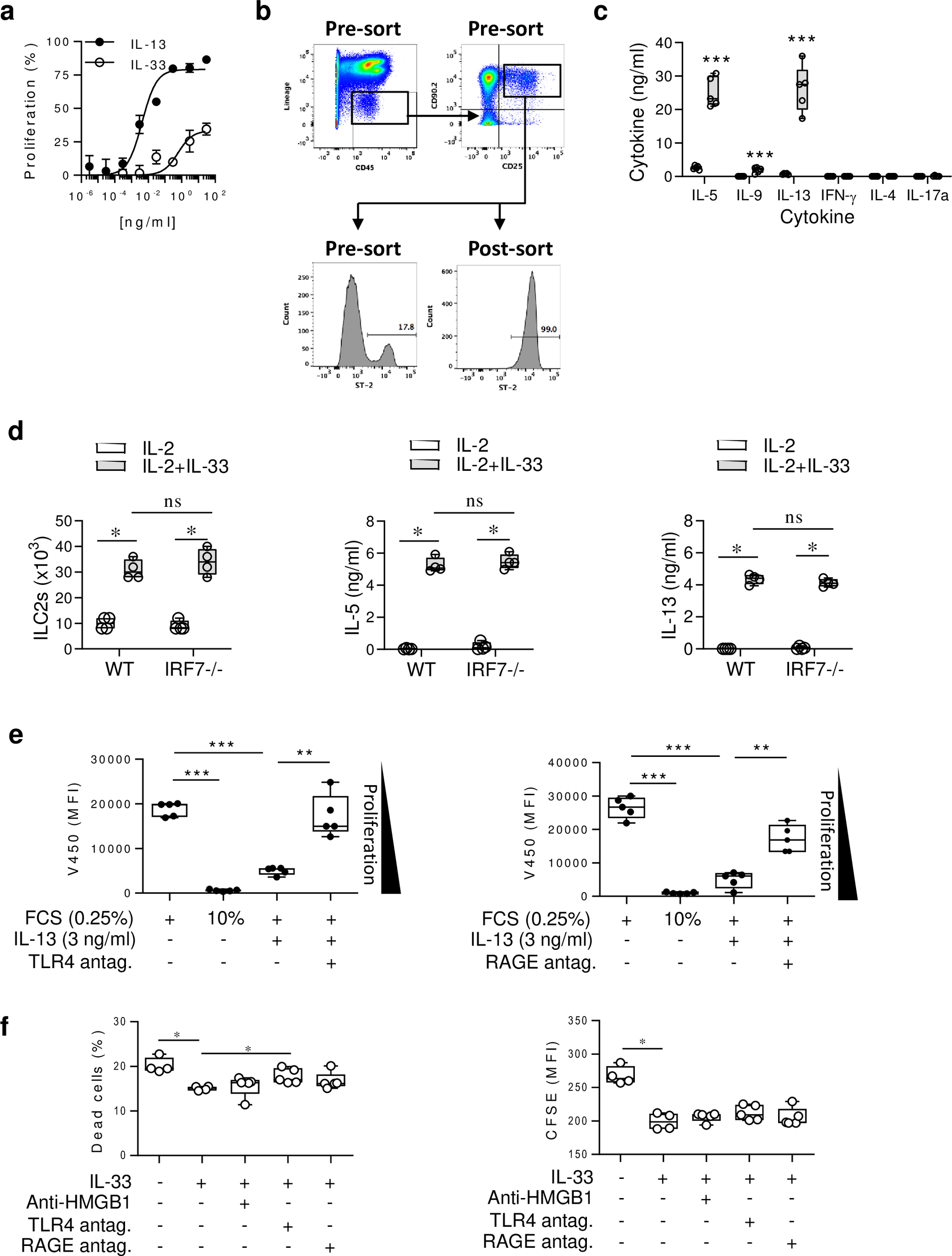

Supplement: S4 Fig — (a) Dose response curve of IL-13 or IL-33 stimulated ASM proliferation. (b) ILC2 purification strategy by FACS; Lineage- gate included CD45R, CD3, CD4, CD11c, CD19, Gr-1, CD11b, CD2, NK1.1, CD49b. The sorted cells were positive for the following four markers: CD90.2+, CD45+, CD25+, and ST2+. (c) IL-2 +/- IL-33 stimulated IL-5, IL-9 IL-13, IFN-γ, IL-4 and IL-17A protein production by ILC2s. (d) ILC2s numbers (left panel), IL-5 (middle panel) and IL-13 (right panel) protein production by lung ILC2s purified from WT or IRF7-/- mice cultured with IL-2 +/- IL-33 (30 ng/ml) for four days. (e) ASM proliferation in response to IL-13 in the presence of LPS-RS (TLR4 antagonist; left) or FPS-ZM1 (RAGE antagonist; right). (f) Percent dead (left) or proliferating ILC2 cells in response to anti-HMGB1 (2G7), TLR4 antagonist (LPS-RS) or RAGE antagonist (FPS-ZM1; right). Data are representative of one experiment, performed twice with similar results. Data is shown as mean ± SEM (a) or as box-and-whisker plots showing quartiles (boxes) and range (whiskers; c-f). Data were analysed by Two-way ANOVA with Tukey post hoc test (a) or T test ((c) or One-way ANOVA with Dunnett post hoc test (d-f); *, P < 0.05; **, P < 0.01; ***, P < 0.001 compared with the respective control group. (TIF) [file ppat.1008651.s004.tif]
