# Supplementary material for: HMGB1 amplifies ILC2-induced type-2 inflammation and airway smooth muscle remodelling
Source: PLoS Pathog. 2020 Jul 13;16(7):e1008651. doi: 10.1371/journal.ppat.1008651 (PMC7377495; doi:10.1371/journal.ppat.1008651)
Supplement: Attachment — Submitted filename: Plos Pathogens Reviewer comments 2020-05-13.pdf [file ppat.1008651.s005.pdf]

EDITOR COMMENT:

Dear A/Prof Phipps,

Thank you very much for submitting your manuscript "HMGB1 amplifies ILC2-induced type-2 inflammation and airway smooth muscle remodelling." for consideration at PLOS Pathogens. As with all papers reviewed by the journal, your manuscript was reviewed by members of the editorial board and by several independent reviewers. The reviewers appreciated the attention to an important topic. Based on the reviews, we are likely to accept this manuscript for publication, providing that you modify the manuscript according to the review recommendations.

Please prepare and submit your revised manuscript within 30 days. If you anticipate any delay, please let us know the expected resubmission date by replying to this email.

When you are ready to resubmit, please upload the following:

[1] A letter containing a detailed list of your responses to all review comments, and a description of the changes you have made in the manuscript.

Please note while forming your response, if your article is accepted, you may have the opportunity to make the peer review history publicly available. The record will include editor decision letters (with reviews) and your responses to reviewer comments. If eligible, we will contact you to opt in or out

[2] Two versions of the revised manuscript: one with either highlights or tracked changes denoting where the text has been changed; the other a clean version (uploaded as the manuscript file).

Important additional instructions are given below your reviewer comments.

Thank you again for your submission to our journal. We hope that our editorial process has been constructive so far, and we welcome your feedback at any time. Please don't hesitate to contact us if you have any questions or comments.

Sincerely,

Mark T. Heise
Section Editor
PLOS Pathogens

Kasturi Haldar
Editor-in-Chief
PLOS Pathogens
orcid.org/0000-0001-5065-158X

Michael Malim
Editor-in-Chief
PLOS Pathogens
orcid.org/0000-0002-7699-2064

AUTHOR REPLY: We thank both the editors and reviewers for their time in reviewing this manuscript. We are pleased that the reviewers recognise the novelty of our findings.

Reviewer Comments (if any, and for reference):

Reviewer's Responses to Questions

Part I - Summary
Please use this section to discuss strengths/weaknesses of study, novelty/significance, general execution and scholarship.

REVIEWER 1 COMMENT: This manuscript by Loh et al utilizes pneumonia virus of mouse (PVM) infection in IRF7-deficient mice, which have impaired anti-viral immunity due to an impaired IFN-alpha response, to understand the role of ILC2 and HMGB1 in viral-induced asthma. A major strength of the paper is the use of a clinically relevant model. PMV replicates several of the features of severe human RSV bronchiolitis, and neonatal animals are used. Furthermore, differential expression of IRF7 may be linked to asthma.

The major findings include:

1. Impaired anti-viral immunity in IRF7-deficient mice is associated with increased epithelial cell injury, increased IL-33 and HMGB1, increased lung ILC2 (the major source of IL-13) and type 2 inflammation, and increased in airway smooth muscle (ASM). Type 2 inflammation is also observed after re-infecting IRF7-deficient mice with PVM and is associated with increased ASM and increased airways hyper-responsiveness (which is modest at best and only observed at the highest methacholine dose). Treatment of IRF7-deficient mice with anti-HMGB1 or anti-IL-33 abrogates lung ILC2 numbers, type 2 inflammation and ASM growth following PVM infection. These findings are important but not surprising.

2. In vitro, IL-13 production by IL-33 treated ILC2 promotes ASM proliferation and ASM expression of TGF-beta and HMGB1. Furthermore, IL-13 acts in an autocrine fashion to increase ILC2 cytoplasmic HMGB1 and release, and HMGB1 in turn stimulates ILC2 (via its receptor RAGE) to produce type 2 cytokines. Treatment of PVM-infected IRF7-deficient mice with a RAGE antagonist decreased type 2 inflammation. Of note, HMBG1-mediated activation of ILC2 has recently been demonstrated in using a mouse model of allergic airway inflammation.

Overall, the manuscript is well written, the results are clear and the topic is of significant interest.

AUTHOR REPLY: We thank the reviewer for their positive comments.

REVIEWER 2 COMMENT: In the current study Phipps and colleagues investigate in the role of HMGB1 in augmenting ILC2 dependent type-2 inflammation in a mouse model of severe respiratory viral infection in early life. The authors that HMGB1 promotes type 2 immunity in a mouse model of severe viral pneumonia, highlighting the promotion of airway smooth muscle. They find this is associated with increases in ILC2, but not Th2, numbers and

ILC2s can directly signal on ASM cells to promote their proliferation through a HMGB1-IL-13-TGFb axis and involves HMGB1's ability to signal through both TLR4 and RAGE. The work builds on previous studies by the group, and in particular, is closely linked to a recent study in the AJRCCM, in which type-2 enhanced inflammation as a result of HMGB1/IRF7 during PVM infection is also described, but the role of epithelial cell death as a mechanism explored (Simpson et al, 2020, PMID 32105156). The two studies therefore do explore distinct experimental mechanisms and the current study provide new insights into the potential mechanisms underlying long term effects of early life viral bronchiolitis with regards to airway remodelling. Overall the study well described and carried out, and the mechanisms underlying viral-induced wheeze and asthma are of both clinical importance and biological interest. There are however a number of areas where additional information or clarification would be beneficial.

AUTHOR REPLY: We thank the reviewer for their positive comments. We address the reviewer's concerns below.

Part II – Major Issues: Key Experiments Required for Acceptance
Please use this section to detail the key new experiments or modifications of existing experiments that should be absolutely required to validate study conclusions.

Generally, there should be no more than 3 such required experiments or major modifications for a "Major Revision" recommendation. If more than 3 experiments are necessary to validate the study conclusions, then you are encouraged to recommend "Reject".

REVIEWER 1 COMMENT: 1. In many of the figures, some of the data points are missing error bars. Is this because the errors are smaller than the symbols used? Or is it because of the number of animals analyzed at a given time point? In other words, when 4-8 animals were used per group, does this mean that 4-8 animals were analyzed at each time point or 4-8 animals for the entire experiment with only 1-2 animals per time point?

AUTHOR REPLY: The number of animals refers to the number of mice at each time point. We have double-checked all of the graphs and the error bars shown are correct. Where an error bar appears to be absent it is because the variation is so low.

REVIEWER 1 COMMENT: 2. Fig 2: In panel (c), no cells are included in the CD25+CD90.2+ ILC2 gate, yet the quantification of IL-13 positive cells on the subsequent panel is 6%. Is this truly representative? It is difficult to assess whether (a) ILC2 in WT are not producing IL-13, (b) there are just fewer numbers of ILC2 in WT mice or (c) there are reductions in both the number of ILC2 and the production of IL-13 by ILC2 in WT mice.

AUTHOR REPLY: Panel b shows that there are fewer ILC2s in the WT mice, and panel c shows that fewer of the ILC2s in the WT mice are producing IL-13. However, we agree with the reviewer that the events in the gate in panel c are hard to see and have therefore substituted this image in the revised manuscript. Thank you for pointing this out.

REVIEWER 2 COMMENT: 1. One seemingly important question arising from the authors work is whether the effects they see on ASM are the result of enhanced type 2 immunity due to IRF7 deficiency are dependent or independent heightened viral loads and damage (such as

necroptosis they have previously reported). Can the authors show the effect of a non-infectious stimuli on ILC2/ASM in IRF7-/- vs WT mice. For instance TLR7 is a potent stimulator of ISGs while intranasal rIL-33 potently promotes ILC2 responses and AHR. This would seem particularly important in the context of segregating the role of HMGB1 on epithelial cells and the effect of ILC2s on ASM.

AUTHOR REPLY: In the mouse model, it is difficult to tease out the precise role of viral load. It is our view that viral load leads to persistent inflammation. This in turn drives immunopathology, necroptosis (as per our recent article in AJRCCM)-associated tissue damage, and alarmin (HMGB1) release. The ensuing alarmin-induced activation of ILC2 promotes type 2 inflammation and tissue remodelling. One cannot easily separate the two events – viral load and type 2 inflammation – as I believe the reviewer is asking. If one were to artificially expose a mouse to supraphysiological doses of alarmins (as proposed) then this would likely recapitulate what we observed in PVM-infected IRF7-/- mice, however, we believe that animal models should seek to simulate gene-environment interactions where this is known to underpin disease. In the co-culture system, we show that IL-33 activated ILC2 induce ASM growth independently of virus infection, and yet this phenotype is HMGB1 dependent. So as described in the paper, HMGB1 works upstream and downstream on ILC2s.

REVIEWER 2 COMMENT: 2. The authors analysis of long term consequences of early life PVM infection are interesting but lack depth. Early life infection with hRSV for instance has been shown to prime Th2 cells capable of eliciting type-2 enhanced disease on either homologous or heterologous rechallenge (e.g. Culley et al, J Exp Med, 2002, PMID 12438429). In the PVM and CRE re-challenge models, can the authors show the relative contribution of ILC2s versus other T cells to this effect? Were the number of each enumerated? In addition, can the authors clarify the why re-challenge with an apparently low dose of PVM fails to elicit infection but promotes prominent inflammation?

AUTHOR REPLY: We agree; the focus of the current paper was understanding the pathogenic mechanisms that promote asthma onset rather than in-depth analyses of the asthma phase. We presume that in the reinfection, the virus is largely neutralised by protective antibodies, at least before the time at which we have sought to detect it. One possible explanation of the inflammatory response is that the rapid virus sensing by innate cells leads to the rapid release of IL-33 and HMGB1. Alternatively (and more likely), is that there are long-lived alterations to the airway mucosa, both in haematopoietic and non-haematopoietic cells. Indeed, we have recently shown that cytoplasmic HMGB1 levels remain high after the primary infection, and that this persists until the time of re-infection or CRE challenge (Lynch et al 2020 Muc Imm). As requested, we have now included ILC2 and T cell numbers in Fig S2e.

REVIEWER 2 COMMENT: 3. TGF-beta is clearly a critical downstream mediator in the authors proposed model, however there seems to be an incomplete assessment of its sources in vivo and activation in vivo. For instance the authors focus on ASM as the relevant source of TGFb however their histology in figure 2F suggests prominent TGFb staining in IRF7-/- mice? This could be particular relevant given that epithelial derived TGF-beta has previously been shown to augment type-2 lung inflammation through increased recruitment of ILC2s (Denney et al, Immunity, 2015, PMID 26588780). Can the authors measured active TGFb in the airways of IRF7-/- versus WT PVM infected mice? Also, although minor, can the authors

also clarify precisely which ELISA they used to identify active, versus latent, TGFb as traditionally this has been challenging via ELISA, and assessment has relied on a bioassays for downstream signalling? Finally in their co-culture system can the authors speculate which cell type is responsible for the activation of TGF-b – do either ILC2s or ASMs express potential activating molecules such as av b6/8, MMP2/9, plasmin.

AUTHOR REPLY: To address the reviewer's point, we have now included new data showing that active TGFβ protein expression is significantly greater in BALF of infected IRF7-/- mice compared to WT mice (Fig 2f). We used the LEGEND MAX™ Free Active TGF-β1 ELISA Kit from Biolegend (#437707). ASM cells are known to secrete TGFβ and can promote its activation via the production of MMPs, plasmin and ROS. Less evidence is available regarding ILC2s. This will need to be explored in future studies.

Part III – Minor Issues: Editorial and Data Presentation Modifications
Please use this section for editorial suggestions as well as relatively minor modifications of existing data that would enhance clarity.

REVIEWER 1 COMMENT: 1. Fig. 1: Given the significant differences in neutrophilic infiltrate, airway epithelial cells sloughing and edema, it would be useful to see representative histology.

AUTHOR REPLY: We do not think that adding the images would add value to the manuscript as the focus of Fig 1 is to provide evidence of HMGB1 (and its isoforms) release in PVM infected mice. These types of images can be found in our other PVM-related manuscripts.

REVIEWER 1 COMMENT: 2. Fig. 1: The time point assessed in panels (h) and (i) should be clarified in the figure or the legend. Additionally, it would be helpful to use the closed vs open circle designation for WT vs IRF7-deficient mice in panel (i) (as is used in the rest of this figure).

AUTHOR REPLY: Thank you for identifying this oversight. We have corrected this in the revised manuscript.

REVIEWER 1 COMMENT: 3. Fig. 1 and S1: The difference in IL-33 in the lung between WT and IRF7-deficient mice following PVM infection (Fig. S1) is more impressive than the differences in the BAL (Fig. 1). This is not surprising as IL-33 is released basolaterally from AEC. I assume BAL fluid is shown in Fig. 1 because only BAL (and not lung) levels are available for the experiments in Fig. 3, 4 and 6?

AUTHOR REPLY: The reviewer's comment is accurate. We include tissue compartment details in all Y axis for clarity.

REVIEWER 1 COMMENT: 4. Fig. S1: In the legend, panel (e) is described as demonstrating IL-4 producing ILC2, CD4 and CD8 T cells. However, the results section

describes this panel only as a quantification of ILC2 in the lung as compared to BAL fluid, without mentioning IL-4 expression, and CD4 and CD8 T cells are not displayed.

AUTHOR REPLY: Thank you for identifying this oversight. We have corrected this in the revised manuscript.

REVIEWER 1 COMMENT: 5. Fig. 2: If the data are available, it would be helpful to see quantification of IL-5 at time points other than day 10.

AUTHOR REPLY: Unfortunately, we did not perform IL-5 ELISA at time points other than day 10.

REVIEWER 1 COMMENT: 6. Fig. 5: It is not clear that panel (a) adds anything to the data shown in panel (b).

AUTHOR REPLY: We agree and have corrected the manuscript accordingly.

REVIEWER 1 COMMENT: 7. Fig. 5: In panel (e), the cytoplasmic HMGB1 staining show is rather subtle. It would be helpful to see a negative control or WT mouse.

AUTHOR REPLY: We have added a representative HMGB1 staining in WT mice in the revised manuscript. Additionally, we have moved the arrows closer to the immunoreactive cells beneath the basement membrane.

REVIEWER 2 COMMENT: 1. The authors should remove all data pertaining to anti-CD25 depletion (Figure S4). While it is understandable that the authors sought to carry out an in vivo experiment evaluating the role of ILC2s in ASM remodelling, and the complexity of ILC2 specific deficient models presents some technical difficulties, the routine use of the same anti-CD25 regime to deplete Tregs (in spite of the surprising failure to have an effect in the lungs in this study) in the majority of other in vivo experiments raises more questions that it resolves. It is also unnecessary for the conclusions drawn from this study.

AUTHOR REPLY: We have now removed the anti-CD25 data as requested.

REVIEWER 2 COMMENT: 2. Figure 1i – can the authors clarify in the figure legend whether the western blot was carried out with or without infection (and at what d.p.i.). If with infection, can the authors confirm IRF7-/- mice have no baseline alteration in the presence of HMGB1 isoforms without infection?

AUTHOR REPLY: We have added the requested details to the figure legend in the revised manuscript. The samples were obtained from infected mice. Regrettably, we are no longer

maintaining the IRF7-/- mice as this project was completed some time ago, so we are unable to address the reviewer's request with respect to naïve samples

REVIEWER 2 COMMENT: 3. Line 159 – remove the work "remarkably".

AUTHOR REPLY: We have corrected this in the revised manuscript.

REVIEWER 2 COMMENT: 4. Line 222 – it would be helpful if the authors could include a line describing the known proliferative effect of FBS on ASM cells in vitro, the mechanism behind this, and its use as a known positive control in this set of experiments.

AUTHOR REPLY: FBS contains various growth factors such as TGFB, so we used 10% FBS as a positive control to induce proliferation (Oida and Weiner, J Immunol Methods, 2010. PMID 20837018).

REVIEWER 2 COMMENT: 5. Fig 2d – the empty and filled symbols are swapped compared to the rest of the figure. (WT empty/KO filled).

AUTHOR REPLY: Thank you for identifying this oversight. We have now corrected this error in the revised manuscript.

REVIEWER 2 COMMENT: 6. Fig 1a. Use of % of AEC stained histologically positive for PVM seems an unconventional measure of viral replication, whole lung analysis using either RT-qPCR or especially traditional plaque assay/TCID50 would seem more appropriate (and account for replication anywhere in the lungs, not only in the airway epithelium).

AUTHOR REPLY: It is not possible to detect PVM in the lungs using a plaque assay. We have attempted this with the support of Dr. Spann (a co-author and experienced virologist) however the loads are too low. We have published several papers (JEM, STM, AJRCCM) using the described method, and demonstrated that the pattern of PVM by IHC is almost identical to qPCR, and prefer IHC since it provides knowledge on virus geography.

OTHER CHANGES:

Line 127-128: In contrast, other known inducers of type-2 inflammation, such as IL-25 and TSLP, were below the limit of detection (data not shown).

REVISED SENTENCE: Line 127-128: In contrast, other known inducers of type-2 inflammation, such as IL-25 and TSLP, were below the limit of detection.

Line 155-156: Other immune cells also expressed dsRed, however their numbers, including that of CD4+ and CD8+ T cells, were negligible (Fig 2d, and data not shown).

REVISED SENTENCE: Other immune cells such CD4+ and CD8+ T cells also expressed dsRed, however their numbers were negligible as compared to ILC2s in infected IRF7-/- mice (Fig 2d).
